# Blood-Brain Barrier Permeability Is Elevated in Type 2 Diabetes and Obesity: Associations with Cognitive Function and Metabolic Markers

**DOI:** 10.21203/rs.3.rs-9698981/v1

**Published:** 2026-05-26

**Authors:** Anujit Saha, Wen Shi, Mohamad N. Elsharydah, Jeff Schaffert, Jack Kaufman, Jennine Leary, Alison Jin, Jaime Almandoz, Hanzhang Lu, Binu P. Thomas

**Affiliations:** University of Texas at Dallas; Johns Hopkins University School of Medicine; University of Texas Southwestern Medical Center; University of Texas Southwestern Medical Center; University of Texas Southwestern Medical Center; University of Texas Southwestern Medical Center; University of Texas Southwestern Medical Center; University of Texas Southwestern Medical Center; Johns Hopkins University School of Medicine; University of Texas Southwestern Medical Center

**Keywords:** Blood-brain barrier, Cognition, Type-2 diabetes, obesity, Alzheimer’s Disease and Related Dementias, Older adults, Preclinical marker, Non-contrast MRI, Executive Function

## Abstract

**Background:**

Type 2 diabetes (T2D) and obesity are established risk factors for Alzheimer’s disease and related dementias, yet the cerebrovascular mechanisms linking metabolic dysfunction to cognitive decline remain poorly understood. Prior human studies relied on gadolinium-based methods to measure blood-brain-barrier (BBB) permeability to larger molecules (~ 550 Da), potentially underestimating subtle BBB disruption. This study aims to assess BBB water permeability in older adults with T2D and obesity using non-contrast WEPCAST MRI, and to evaluate associations between BBB integrity, metabolic markers, and cognitive function.

**Methods:**

Twenty-eight older adults − 12 with T2D and obesity (BMI ≥ 30 kg/m^2^) and 16 age- and sex- matched controls - underwent WEPCAST MRI to quantify the BBB permeability measures: permeability-surface area product (PS), water extraction fraction (E) and global cerebral blood flow (CBF), along with T1-Weighted structural MRI acquisition. Cognitive function and fasting blood biomarkers were assessed in all participants. Associations between PS versus metabolic and cognitive function were examined using linear regression adjusted for age and sex. An additional adjustment for statin use was made for hemoglobin A1c (HbA1c) and lipid markers.

**Results:**

T2D participants exhibited significantly elevated BBB water permeability (PS: P = 0.001, Hedges’ g = 1.10) despite preserved CBF. Specifically, higher PS was associated with HbA1c (f^2^ = 0.28, P = 0.016) and lower circulating cholesterol (total cholesterol: f^2^ = 0.49, P = 0.003; LDL: f^2^ = 0.45, P = 0.004; HDL: f^2^ = 0.18, P = 0.053). The T2D group demonstrated lower scores across multiple domains including memory, attention, learning, executive function, and processing speed (g = 0.80–1.42). Higher PS was associated with poorer measures of executive function (P-values < 0.05).

**Conclusions:**

Older adults with T2D and obesity exhibit elevated BBB water permeability, detectable by non-contrast WEPCAST MRI. Higher BBB water permeability was associated with higher glycemic burden and lower executive function, providing preliminary evidence that BBB permeability may serve as a novel imaging biomarker linking metabolic dysregulation to early cognitive vulnerability in T2D that follows a traditional “frontal-subcortical” pattern.

## BACKGROUND

Type-2 diabetes (T2D) affects over 40 million Americans and more than 500 million people worldwide, with an annual healthcare burden exceeding $400 billion in the United States alone[1]. Beyond its well-known metabolic consequences, T2D substantially increases the risk of developing Alzheimer’s disease and related dementias (ADRD)[2, 3]. While T2D and AD share overlapping pathophysiology, including insulin resistance, chronic neuroinflammation, oxidative stress, and cerebrovascular dysfunction [2, 4], the specific mechanisms by which T2D worsens cognitive decline in older adults remain unclear.

Neuropsychological deficits in T2D most consistently affect processing speed, attention, executive function, and memory [2]. However, the severity and profile vary considerably across individuals, reflecting multifactorial underlying mechanisms including chronic hyperglycemia, vascular injury, and metabolic dysregulation [5, 6]. Among these domains, attention, speed, and executive functions are of particular interest given their relation to cerebrovascular compromise [7, 8]. Meta-analytic evidence demonstrates that cognitive flexibility and inhibitory control are among the earliest and most consistently impaired functions in subcortical vascular cognitive decline [9]. Importantly, the underlying mechanisms that affect these specific cognitive domains in clinically unimpaired older adults remain poorly characterized.

One potential pathway involves disruption of the blood-brain-barrier (BBB), a selectively permeable interface formed by endothelial cells, vascular tight junctions, pericytes and astrocytes within the neurovascular unit. It is critical for maintaining the brain’s microenvironment and shielding neural tissue from circulating toxins [4, 10]. Preclinical work has firmly established that the metabolic impact of T2D, namely chronic hyperglycemia, insulin resistance, and dyslipidemia, disrupts BBB integrity through oxidative stress, tight junction protein degradation, and pericyte dysfunction [4, 11]. Notably, the most widely used preclinical models of T2D -including leptin knockout obese db/db mice [11] and high fat induced diabetic rodents - inherently combine hyperglycemia with obesity, reflecting the clinical reality that these conditions frequently co-occur [12]. In these models, obesity-driven systemic inflammation, insulin resistance, and pericyte loss act synergistically with hyperglycemia to disrupt the BBB [13]. However, this combined effect of T2D and obesity on BBB permeability has not been examined in humans.

In parallel, human neuroimaging studies have identified BBB breakdown as an early biomarker for cognitive decline and AD, with some evidence suggesting it precedes amyloid and tau pathology [4, 14]. Metabolic factors central to T2D, such as glycemic burden may directly modulate BBB permeability, with emerging animal studies also suggesting that cholesterol and HDL may play protective roles at the barrier [15]. If BBB dysfunction mediates the link between T2D and cognition, one would expect BBB permeability to correlate with both metabolic markers and cognitive performance. However, this has not been tested in human T2D and obesity studies.

Despite strong preclinical evidence that hyperglycemia disrupts BBB tight junctions and increases permeability [11, 16], human evidence remains limited and inconsistent. Reviews of experimental and clinical literature have highlighted contradictory findings, with the BBB reported as either intact or variably leaky in diabetes depending on the tracer, model, and methodology used [17]. The few human neuroimaging studies that exist have important limitations. Starr et al. [18] first reported increased gadolinium enhancement in the basal ganglia of 10 well-controlled T2D patients with significantly greater vascular burden evidenced by white matter lesion volume, so it remains unclear whether the increased permeability is a direct effect of diabetes or concurrent cerebrovascular disease. A more recent study by Qiao et al. [19] used DCE-MRI to compare BBB permeability between T2D patients with and without established cognitive impairment with Montreal Cognitive Assessment (MoCA) scores of ~ 19 vs. ~27 respectively, showing elevated K-trans in the cognitively impaired subgroup. However, this design cannot address whether BBB disruption occurs in the earlier, pre-clinical stages of cognitive decline in T2D. Importantly, all prior studies [18–20] required intravenous gadolinium administration and measured permeability to a relatively large contrast molecule (~ 550 Da), which may underestimate subtle barrier dysfunction, and is contraindicated in renal impairment common among older adults with T2D.

Water is a small endogenous molecule (18 Da) whose permeability across the BBB may be more sensitive to early microvascular changes than large contrast agents [21]. Water Extraction with Phase-Contrast Arterial Spin Tagging (WEPCAST) MRI is a non-contrast technique that quantifies BBB permeability to water by measuring the fraction of arterially labeled spins that drain into the superior sagittal sinus [21, 22]. WEPCAST has been validated against contrast-based methods (R = 0.73) [23], with an inter-visit reproducibility of 5% [22].

Previous research using this technique has demonstrated that individuals with mild cognitive impairment (MCI) exhibit significantly elevated BBB water permeability compared to age- and sex-matched controls [24]. Crucially, this study identified a dissociation between molecular scales of barrier breakdown. While increased permeability to water was strongly associated with poorer cognitive performance and Alzheimer’s disease biomarkers (Aβ and p-tau), no such association was found for larger molecules like albumin. This suggests that non-contrast water permeability measures capture early, functionally relevant microvascular changes that precede macromolecular leakage. These findings establish BBB water permeability as a sensitive biomarker for detecting preclinical BBB dysfunction, supporting its application in investigating early microvascular compromise in people with T2D.

This study is the first to assess BBB permeability to water in older adults with T2D and obesity using this novel WEPCAST MRI. We hypothesized that participants with T2D would exhibit elevated BBB permeability and lower cognitive performance compared to age- and sex-matched cognitively intact controls. We further hypothesized that BBB permeability would be associated with metabolic markers and with cognitive function, consistent with BBB dysfunction serving as a mechanistic link between metabolic dysregulation and cognitive decline in T2D.

## METHODS

### Participants

This study involved older adults recruited from the clinics at the University of Texas Southwestern (UTSW) Medical Center to examine the impact of T2D and obesity on brain health. The study was approved by the UTSW Institutional Review Board, and all participants provided written informed consent prior to participation.

Inclusion criteria for older adults with T2D and obesity were: 1) clinical diagnosis of T2D; 2) Hemoglobin A1c (HbA1c) ranging between 6–10% over the six months pre-enrollment; 3) BMI of 30–45 kg/m^2^; and 4) controlled hypertension. For control group the criteria included: 1) HbA1c less than 5.7% within the past 6 months; 2) controlled hypertension; and 3) BMI of 22.5–27.5 kg/m^2^. Exclusion criteria included 1) MRI contraindications; 2) major neurological (including stroke, transient ischemic attack, and neurocognitive disorders) or psychiatric conditions, 3) prior GLP-1 receptor agonist or insulin use within six months; 4) prior bariatric surgery; 5) active smoking; 6) body weight > 450 pounds; 7) prior heart disease (e.g., arrhythmia, coronary artery disease, pericarditis, or angina); 8) pre-menopausal status; 9) weight loss > 5% within the preceding 6 months; 10) clinically significant age-related hearing loss and 11) chronic kidney disease (stage ≥ 4). Controls were additionally excluded for prior cardiovascular disease.

### MRI Acquisition

All imaging was performed on a 3T Siemens Prisma scanner (Siemens Healthineers, Erlangen, Germany) at the Advanced Imaging Research Center (AIRC), UTSW Medical Center. The body coil was used for RF transmission, and a 64-channel head coil was used for signal reception. To minimize motion during scans, cushions and/or inflatable pads were placed around each participant’s head inside the head coil. No contrast agents were administered.

**Figure 1:**
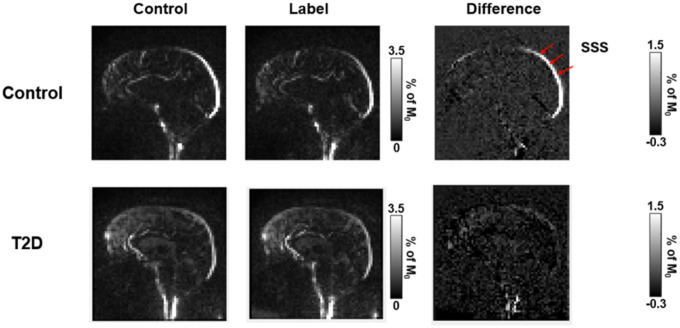
Representative WEPCAST MRI images (Control, Label, and Difference) from one T2D and one control participant, illustrating BBB permeability measurement at the superior sagittal sinus (SSS). Red arrows indicate the SSS region where the venous signal is acquired.

BBB permeability to water was measured using WEPCAST MRI, a non-contrast technique that quantifies the fraction of arterially labeled water that crosses the BBB into brain tissue [21, 22]. Briefly, a pseudo-continuous arterial spin labeling (pCASL) module is applied at the cervical spine to invert spins in the feeding arteries. A portion of the labeled water crosses the BBB and is extracted into the tissue, whereas the unextracted spins drain into the cerebral veins. By measuring the residual labeled signal in the superior sagittal sinus (SSS) (Fig. 1) using flow-encoded phase-contrast acquisition, the global water extraction fraction (E) is estimated. WEPCAST data were acquired in a 10 mm thick midsagittal plane (voxel size = 3.1 × 3.1 × 10 mm^3^, TR/TE = 9200/9.5 ms, labeling duration = 4 s, post-labeling delay = 3 s, VENC = 20 cm/s, GRAPPA = 3, 10 control/label pairs, scan duration ≈ 8 min). A proton density weighted (M0) image was also acquired for signal calibration. Background suppression was applied to minimize tissue signal contamination.

Global cerebral blood flow (CBF) was quantified using phase-contrast (PC) MRI at four feeding arteries (left and right internal carotid arteries, left and right vertebral arteries) [25]. PC MRI scan used the following two parameters: TR = 25 ms, TE = 8 ms, FOV = 200 × 200 mm^2^, voxel size = 0.5 × 0.5 × 5 mm^3^, flip angle = 15◦, VENC = 40 cm/s. Total cerebral blood flow was computed as the sum of flow across the four arteries and normalized by brain volume to yield global CBF in the units of mL/100g/min.

### MRI data analysis

WEPCAST MRI data were processed using a generalized venous signal (GVS) model [22], which simultaneously estimates E and venous transit time (VTT) from both control and labeled WEPCAST signals. VTT is a physiological measure of the blood transit time from the capillaries to the SSS, which can be independently measured using other techniques [26].

The BBB permeability-surface area product (PS) was computed from E and global cerebral blood flow (f) using the Renkin-Crone Eq. (1)[27]:

(1) PS=−ln(1-\varvecE)⋅f

Individual hematocrit values from blood were used to determine the blood T1 value for each participant [28]. All WEPCAST scans were visually inspected for motion artifacts, and participants with excessive motion were excluded.

Additionally, T1-weighted multi echo magnetization prepared rapid gradient echo (MEMPRAGE) scan was acquired with 1.0 mm^3^ isotropic resolution for brain volumetry and structural analysis. Computational Anatomy Toolbox (CAT12)[29] was used to compute total brain volume.

WEPCAST and PC-MRI data were processed in MATLAB (MathWorks, Natick, MA).

### Blood Biomarkers

Fasting blood samples were drawn from each participant and processed by Quest Diagnostics. HbA1c and fasting glucose characterized glycemic control. Fasting insulin was used to compute HOMA-IR [30]. A standard lipid panel including total cholesterol, HDL, LDL (calculated), triglycerides, and non-HDL cholesterol was also measured. Statin use was recorded from participant medication lists.

### Neuropsychological Assessment

Standard neuropsychological testing was performed on the same day as the imaging visit by trained neuropsychologists or psychometricians at UTSW Medical Center. The battery included the Repeatable Battery for the Assessment of Neuropsychological Status (RBANS), the Delis-Kaplan Executive Function System (D-KEFS) Color-Word Interference Test, Trail Making Tests A and B (TMT-A, TMT-B), the Controlled Oral Word Association Test (FAS), Animal Fluency, and the Boston Naming Test (BNT-30). RBANS yields age-normed percentile ranks and scaled scores across domains including immediate memory, attention, processing speed, language, visuospatial function, delayed memory, and global cognition. D-KEFS test percentiles are similarly age-normed, while TMT-A, TMT-B, FAS, and Animal Fluency raw scores are normed for age, sex and education.

### Statistical Analysis

Normality of continuous variables was assessed using the Shapiro-Wilk test. Welch’s t-test was used for group comparisons as it is robust to mild departures from normality. For categorical variables, Fisher’s exact test was used. For variables with substantial skewness, results were confirmed using non-parametric Mann-Whitney U tests.

Hedges’ g was reported as a bias-corrected effect size for all group comparisons. Hedges’ g generally follows Cohen’s benchmarks of effect sizes where 0.2 is considered small, 0.5 medium, and 0.8 or higher large. Group differences in BBB and hemodynamic measures were tested using Ordinary Least Squares (OLS) regression adjusting for age and sex where the p-values for these measures reflect the group coefficient from the adjusted model.

Associations between PS and metabolic markers were also examined using OLS regression adjusting for age, sex, and statin usage. Statin use was included as a covariate in all metabolic models because of its impact on glycemic levels and lipid-glucose metabolism [31, 32]. For associations between PS and cognitive performance, OLS regression was adjusted for covariates to match the normative adjustments applied by each cognitive measure.

For each model, the partial R^2^ attributable to the predictor was computed, and Cohen’s f^2^ derived as partial R^2^ / (1 − model R^2^), where ≥ 0.02, ≥ 0.15, and ≥ 0.35 correspond to small, medium, and large effects. Unstandardized β coefficients are reported with 95% CIs. Significance was set at P < 0.05. Statistical analyses were performed in Python 3 (statsmodels, scipy).

## RESULTS

### Participant Characteristics

The study involved 32 participants in two age and sex-matched groups: T2D (N = 14) and control (N = 18). Quality assessment of the WEPCAST MRI data from all participants revealed severe motion artifacts from two participants per group. Following exclusion of WEPCAST data from these participants, the imaging analysis included 16 controls and 12 T2D participants (N = 28). Demographic and clinical characteristics of these participants are summarized in Table 1.

Groups were well matched for age, sex distribution, and years of education (all P > 0.05). An additional control participant was excluded from neuropsychological analyses (N = 27) due to a language barrier that affected test performance.

Most measures, including the primary outcomes such as PS and E, were found to be normally distributed within each group. Participants with T2D exhibited significantly higher BMI and waist-to-height ratio, reflecting greater central adiposity (all P < 0.001). Markers of glycemic dysfunction, including HbA1c and HOMA-IR, were also elevated in the T2D group (all P ≤ 0.05). Mean arterial pressure did not differ between groups.

Among blood lipid measures, total cholesterol, HDL, and LDL were lower in the T2D group (all P ≤ 0.05) (Table 1). Statin use was more common in the T2D group, with 9 out of 12 participants (75%) taking statins compared with 7 of 16 controls (44%), though this difference did not reach statistical significance (P = 0.136).

### BBB Permeability and Brain Hemodynamic Measures

**Figure 2:**
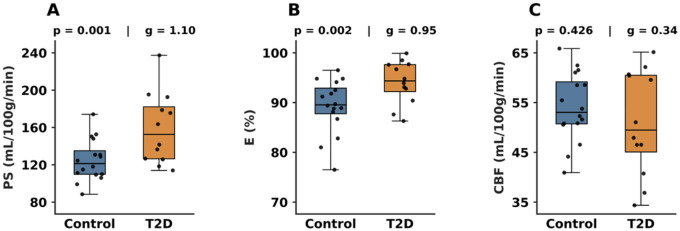
(A) Permeability-surface area product (PS), (B) water extraction fraction (E), and (C) global cerebral blood flow (CBF) differences between T2D and Control groups. P-values are from OLS regression adjusted for age and sex. Hedges’ g is reported as a bias-corrected effect size.

Group differences in BBB and hemodynamic measures are shown in Table 2 and Fig. 2. Consistent with the representative images in Fig. 1, the T2D group showed a significantly lower WEPCAST difference signal in the SSS (p = 0.027), suggesting that the majority of arterially labeled water has been extracted into tissue, indicating a more permeable BBB. Quantitatively, participants with T2D demonstrated significantly elevated BBB permeability to water compared to controls. Specifically, PS was higher in the T2D group (P = 0.001, g = 1.10), representing a large effect size. The water extraction fraction (E) was similarly higher (P = 0.002, g = 0.96) in the T2D group, while global cerebral blood flow (CBF) did not differ between groups. This provided evidence that the higher measured BBB permeability was driven by the elevated water extraction fraction at the BBB interface.

VTT was not significantly different between groups. Together, these results indicate that T2D is associated with increased BBB permeability independent of global cerebral perfusion.

### Associations Between BBB Permeability and Metabolic Markers

**Figure 3:**
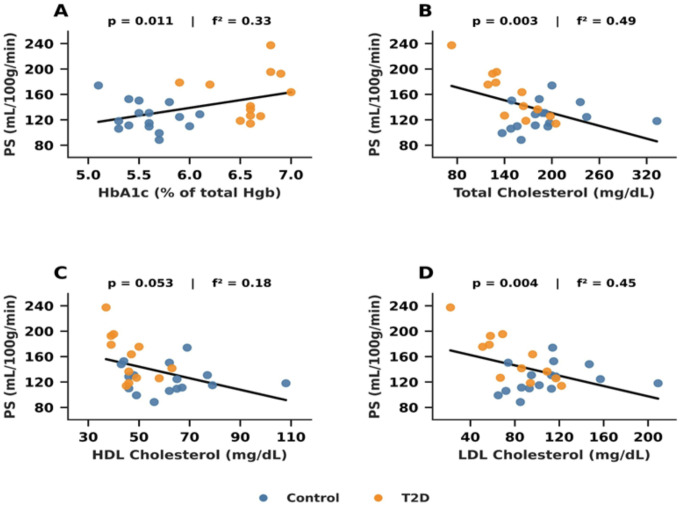
Associations between BBB permeability-surface area product (PS) and metabolic markers: (A) HbA1c, (B) total cholesterol, (C) HDL cholesterol, and (D) LDL cholesterol. Regression lines represent unadjusted bivariate fits for visualization. Cohen’s f^2^ (effect size) and P-values are from OLS regression adjusted for age, sex, and statin use.

PS was positively associated with HbA1c (f^2^ = 0.33, P = 0.011), suggesting that higher glycemic burden is associated with greater BBB permeability (Table 3, Fig. 3). Inverse associations were observed between PS and total cholesterol (f^2^ = 0.49, P = 0.003), LDL (f^2^ = 0.45, P = 0.004), and HDL (f^2^ = 0.18, P = 0.053).

Additionally, HOMA-IR showed a positive association with water extraction fraction (f^2^ = 0.19, P = 0.053), suggesting that insulin resistance may contribute to BBB water permeability beyond glycemic burden as captured by HbA1c.

### Cognitive Function

**Figure 4:**
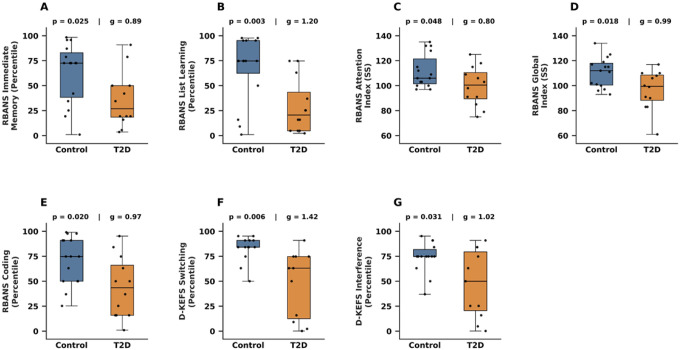
Group differences in cognitive performance between T2D and Control subjects. (A) RBANS Immediate Memory, (B) RBANS List Learning, (C) RBANS Attention Index, (D) RBANS Global Index, (E) RBANS Coding, (F) D-KEFS Switching, and (G) D-KEFS Interference. P-values are reported from Welch’s t-test. Hedges’ g is reported as a bias-corrected effect size.

The T2D group scored lower across multiple cognitive tests with large effect sizes (Table 4, Fig. 4). Significant differences were observed for individual tests of RBANS List Learning (P = 0.003, g = 1.20), a measure of verbal learning and RBANS Coding (P = 0.020, g = 0.97) reflecting attention, processing speed, and visuomotor co-ordination. Significant differences were also observed for domain-associated tests of RBANS Immediate Memory (P = 0.025, g = 0.89), RBANS Attention (P = 0.048, g = 0.80), and RBANS Global Index (P = 0.018, g = 0.99). Cognitive flexibility, measured by the D-KEFS Switching (P = 0.006, g = 1.42) and D-KEFS Interference (P = 0.031, g = 1.02), was significantly lower in the T2D group. A trend-level difference with medium effect size was found in TMT-B (P = 0.066, g = 0.74), reflecting poorer executive functioning in the T2D group. In contrast, no significant group differences were observed for RBANS Visuospatial (P = 0.157, g = 0.58), Delayed Memory (P = 0.162, g = 0.54), FAS (P = 0.123, g = 0.62), or Language percentiles (P = 0.111, g = 0.63).

### Association Between BBB Permeability and Executive Function

**Figure 5:**
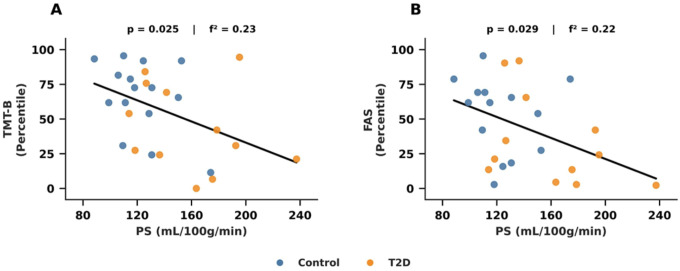
Association between BBB permeability (PS) and cognitive performance. (A) Trail Making Test B (TMT-B), and (B) Controlled Oral Word Association Test (FAS), both shown as percentile. P-values and Cohen’s f^2^ (effect size) are adjusted for age, sex and education.

To examine whether BBB permeability was associated with cognitive performance, PS was tested against measures of executive function given that executive dysfunction is a hallmark of subcortical vascular cognitive impairment and cerebrovascular disease [9]. Of these, TMT-B (f^2^ = 0.23, P = 0.025) and FAS (f^2^ = 0.22, P = 0.029) showed significant inverse associations with PS, indicating that higher BBB permeability was linked to poorer executive function (Table 3, Fig. 5). Other executive function measures - D-KEFS Switching (f^2^ = 0.16, P = 0.071), D-KEFS Interference (f^2^ = 0.08, P = 0.193) - showed similar inverse associations but did not reach statistical significance.

## DISCUSSION

This study is the first to assess BBB permeability to water in older adults with T2D using non-contrast WEPCAST MRI. Our findings reveal three key observations. First, T2D participants exhibited significantly elevated BBB permeability (PS) compared to age- and sex-matched controls, despite preserved global cerebral blood flow. Second, BBB permeability was associated with HbA1c and lipid profiles, providing evidence that chronic glycemic dysregulation contributes to microvascular barrier dysfunction while higher circulating cholesterol was associated with lower BBB permeability, consistent with a potential protective role. Third, higher BBB permeability was associated with poorer executive function, suggesting that BBB dysfunction may represent a mechanistic link between metabolic and frontal-subcortical cognitive decline observed in the T2D group.

### Elevated BBB permeability to water in T2D and obesity: a novel human finding

The elevation in BBB permeability to water observed in the T2D and obesity group represents a large effect that was detectable in a modest sample of 28 participants. These findings are consistent with preclinical models of T2D and obesity, in which hyperglycemia drives tight junction protein loss, neuroinflammation, pericyte dysfunction, collectively compromising BBB integrity [11, 13, 16]. Our study mirrors this clinically relevant phenotype by enrolling T2D participants with co-existing obesity and demonstrates for the first time that the combined metabolic burden translates to elevated BBB water permeability in humans.

Our findings also extend and clarify previous human observations. Starr et al. [18] reported increased gadolinium enhancement in T2D patients, but this finding was confounded by greater white matter lesion burden in the diabetic group, leaving open whether the increased permeability was attributable to diabetes itself or to concurrent cerebrovascular disease. In our cohort, participants with established neurological conditions were excluded, minimizing the potential confounding from overt cerebrovascular disease. Similarly, while Qiao et al. [19] demonstrated elevated K-trans in T2D patients with established cognitive impairment (MoCA ~ 19), our study shows that BBB water permeability is already elevated in cognitively intact older adults with well-controlled T2D (mean HbA1c 6.6%), suggesting that early BBB dysfunction is detectable at a pre-clinical stage before overt cognitive impairment manifests. Moreover, by using water (18 Da) rather than gadolinium (~ 550 Da) as the permeability tracer, the present approach may capture earlier and more subtle barrier changes that precede the macromolecular leakage detected by DCE-MRI.

An important aspect of our results is that the elevated PS occurred in the absence of group differences in global cerebral blood flow. The absence of a PS-CBF association confirms that PS captures flow-independent BBB permeability. This validation is critical for interpreting the clinical relevance of PS as a biomarker of microvascular integrity rather than a hemodynamic artifact.

The positive association between HbA1c and PS suggests that higher glycemic burden is linked to poorer BBB integrity, consistent with established mechanisms by which prolonged hyperglycemia damages the BBB[11, 16, 33]. The positive association between HOMA-IR and water extraction fraction (E) further suggests that insulin resistance may contribute to BBB dysfunction. Insulin signaling at the brain endothelium regulates tight junction assembly, glucose transport and endothelial homeostasis [34–37], and impaired insulin receptor function in cerebral microvasculature has been linked to both T2D and Alzheimer’s disease [38]. Together, these findings indicate that multiple facets of metabolic dysregulation – both glycemic and insulin-related – may compromise barrier integrity.

Beyond glycemic control, our blood lipid findings provide mechanistic insight into the structural and functional determinants of BBB integrity. The inverse association between HDL and PS is consistent with evidence that HDL exerts protective effects on the vascular endothelium, including the promotion of endothelial nitric oxide signaling [39], suppression of NF-κB mediated vascular inflammation and adhesion molecules [40], and stabilization of tight junction complexes to reduce permeability [41]. Clinically, higher circulating HDL has been associated with a lower prevalence of T2D in large cohorts, including NHANES [42], supporting the broader notion that more favorable lipid profiles may confer cerebrovascular protection, including at the BBB.

The inverse associations observed between LDL, total cholesterol, and PS require careful interpretation considering both BBB biology and emerging large-scale epidemiological data. Cholesterol is a fundamental structural component of endothelial cell membranes and lipid rafts, which are critical for tight junction organization and transcellular transport regulation [8]. Experimental evidence indicates that depletion of membrane cholesterol can destabilize tight junctions and increase BBB permeability [43], suggesting that simply lowering circulating cholesterol in T2D may not directly translate to improved barrier integrity. Population-based and genetic studies further demonstrate that lower circulating LDL is associated with a higher risk of T2D. In a large UK Biobank study [44], circulating LDL showed a strong inverse association with T2D prevalence, and genome-wide analyses identified multiple loci where LDL lowering alleles concurrently increase T2D risk, many of which are linked to hepatic lipid handling. Complementary work by Feng et al. [45] in a large EHR cohort showed that very low LDL concentration in the absence of statin therapy (≤ 60 mg/dL) was associated with approximately two-fold higher odds of T2D compared with normal LDL (90–130 mg/dL) with consistent effects across sex and BMI strata. Together, these findings support the interpretation that low LDL is associated with T2D, suggesting that it frequently co-occurs with metabolic dysfunction, such as impaired insulin signaling, that drives BBB damage [38, 46, 47].

Statin therapy further complicates the interpretation of lipid-BBB relationships in clinical cohorts. Statin use is associated with an increased risk of incident T2D, likely through effects on insulin sensitivity and β-cell function [31]. In individuals with T2D taking statins, lower LDL and total cholesterol commonly reflect pharmacologic LDL lowering on a background of substantial cardiometabolic burden. Despite these lower circulating LDL levels, the T2D group exhibited significantly higher BBB permeability, suggesting that statin-induced cholesterol reduction does not offset the BBB burden associated with hyperglycemia and insulin resistance.

Taken together, our metabolic findings point to a delicate homeostatic balance between glycemic and lipid pathways in maintaining BBB integrity. Chronic hyperglycemia appears to cause damage to BBB integrity, as indicated by its increased permeability to water, while adequate circulating cholesterol may be necessary to maintain endothelial membrane structure and tight junction stability. When this balance is disrupted, as in T2D where hyperglycemia coexists with dyslipidemia and pharmacological lipid lowering, the BBB may become increasingly vulnerable. This dual vulnerability with glycemic injury and insufficient lipid-mediated structural support may explain why T2D confers such elevated risk for cerebrovascular pathology and cognitive decline. These observations are hypothesis-generating, and larger longitudinal studies will be required to disentangle the independent and interactive contributions of glycemic and lipid pathways to BBB integrity in T2D.

### BBB permeability and cognitive decline

Despite being cognitively intact by clinical criteria, the T2D group demonstrated significantly lower cognitive function across neuropsychological measures spanning verbal learning, attention, processing speed, executive function, and global cognition (g = 0.80–1.42 for significant comparisons). The pattern of affected domains is consistent with a frontal-subcortical cognitive profile, which is commonly observed in cerebrovascular diseases [9, 48]. Notably, visuospatial, delayed memory, and language abilities were relatively spared, consistent with a vascular rather than purely neurodegenerative mechanism for the observed cognitive differences in our study.

Moreover, the associations between PS and measures of executive function such as TMT-B and FAS provide preliminary evidence that BBB water permeability may serve as a biomarker linking metabolic dysfunction to decline in executive function. Increased BBB water permeability may be indicative of vascular insult to the frontal-subcortical networks, which is often implicated in conditions such as small vessel disease [48].

This vulnerability may be compounded by co-occurring obesity, which has independently been shown to impair cerebrovascular reactivity and cognition, with improvements observed following weight loss [49, 50].

Taken together with the metabolic marker associations, these findings suggest that glycemic burden and altered lipid metabolism may compromise BBB integrity, contributing to the frontal-subcortical cognitive vulnerability observed in cognitively intact older adults with T2D and obesity. A future study with larger number of participants is needed to be better able to account for mechanistic association.

## LIMITATIONS

Several limitations should be considered. First, the cross-sectional design precludes causal inference as the observed associations between PS, metabolic markers, and cognition may reflect shared underlying pathology rather than a causal chain. Longitudinal studies are needed to determine whether BBB permeability changes precede or follow cognitive decline in T2D.

Moreover, WEPCAST provides a global measure of BBB permeability at the SSS rather than regional estimates which cannot capture regional disruption relevant to the observed cognitive deficits. The sample size (n = 28) limits statistical power, particularly for the regression analyses. Several metabolic and cognitive associations approaching significance may reach conventional thresholds in larger cohorts. However, the consistently large effect sizes (g > 0.80 for group differences; f^2^ = 0.22–0.49 for regressions) provide confidence that the observed effects are not artifacts of sampling variability.

The high prevalence of statin use in the T2D group complicates the interpretation of metabolic and PS associations. Although we adjusted for statin use in all metabolic associations, dosage and duration of statin use were not accounted for. Additionally, participants were recruited from a single center and were predominantly well-educated, which may limit generalizability to more diverse populations. Multiple comparison corrections, for metabolic and cognitive associations, were not performed due to the exploratory nature of this study. The results should therefore be interpreted as hypothesis-generating, and replication in larger cohorts is warranted.

Finally, white matter hyperintensity (WMH) burden, a standard marker of cerebrovascular disease, was not quantified in the present study. Although participants with neurological conditions were excluded at enrollment, subclinical white matter lesion burden may still differ between groups and could contribute to the observed PS-cognition associations. Future studies incorporating FLAIR imaging and WMH quantification are warranted to disentangle the contributions of BBB disruption and white matter injury to frontal-subcortical cognitive vulnerability in T2D.

## CONCLUSION

This study provides the first evidence that older adults with T2D and obesity exhibit elevated BBB permeability to water, as measured by non-contrast WEPCAST MRI. BBB permeability was linked to chronic glycemic burden and to executive function, suggesting that microvascular barrier dysfunction may represent an early and measurable pathological mechanism connecting metabolic dysregulation to cognitive decline in T2D. These findings support the development of BBB water permeability as a non-invasive imaging biomarker for monitoring cerebrovascular health in T2D and highlight the potential importance of glycemic control and lipid homeostasis in preserving BBB integrity. The non-contrast nature of WEPCAST MRI makes it particularly well-suited for longitudinal monitoring of BBB integrity in T2D, a population in which renal comorbidities frequently preclude repeated gadolinium administration. Larger longitudinal studies incorporating both WEPCAST MRI and circulating BBB biomarkers are warranted to determine whether BBB dysfunction is a modifiable target for reducing ADRD risk in the T2D population.

## Figures and Tables

**Table 1:** Demographic and clinical characteristics of T2D and control groups.

	T2D (N = 12)	Control (N = 16)	P	Hedges’ g
**Demographics**				
Age (years)	69.8 ± 6.5	66.9 ± 6.9	0.269	0.44
Sex (male/female)	5/7	7/9	1.000	—
**Diabetes markers**				
HbA1c (%)	6.6 ± 0.3	5.6 ± 0.3	< 0.001*	3.45
HOMA-IR (a.u.)	5.8 ±3.1	2.4 ± 1.8	0.004*	1.35
**Anthropometries**				
BMI (kg/m^2^)	32.8 ± 2.5	24.3 ± 2.4	< 0.001*	3.37
Waist/height ratio	0.67 ± 0.06	0.52 ± 0.05	< 0.001*	2.58
**Lipids**				
Total cholesterol (mg/dL)	149.5 ± 37.5	192.3 ± 47.7	0.013*	−0.95
HDL (mg/dL)	46.6 ± 7.8	61.6 ± 17.0	0.005*	−1.05
Triglycerides (mg/dL)	151.7 ± 76.4	109.2 ± 60.9	0.128	0.61
LDL (mg/dL)	79.0 ± 30.1	109.8 ± 36.5	0.022*	−0.88
Non-HDL (mg/dL)	102.9 ± 33.8	130.7 ± 39.2	0.056	−0.73
**Medications**				
Statin use, n (%)	9 (75%)	7 (44%)	0.136	—
**Vitals**				
SBP (mmHg)	133.3 ± 20.6	124.5 ± 11.0	0.198	0.54
MAP (mmHg)	96.3 ± 10.9	91.2 ± 7.3	0.171	0.56

* P < 0.05.

Values are mean ± SD unless otherwise noted. Group comparisons used Welch’s t-test for continuous variables and Fisher’s exact test for categorical variables. Hedges’ g reported for continuous variables (T2D − Control). Effect size not computed for sex or statin use.

SBP = systolic blood pressure; MAP = mean arterial pressure; WMHV = white matter hyperintensity volume; TIV = total intracranial volume.

**Table 2:** BBB permeability and global cerebral blood flow (CBF) measures.

PS (mL/100g/min)	T2D (N = 12)	Control (N = 16)	P	Hedges’ g
	158.8 ± 38.0 [134.6, 183.0]	124.8 ± 22.5 [112.8, 136.8]	0.001*	1.10
E (%)	94.1 ± 4.3 [91.4,96.9]	89.2 ± 5.4 [86.3,92.1]	0.002*	0.96
CBF (mL/100g/min)	51.0 ± 10.5 [44.3, 57.7]	54.0 ± 7.0 [50.3, 57.8]	0.426	−0.34

* P < 0.05, adjusted for age and sex.

Values are mean ± SD with 95% confidence intervals. P-values are from OLS regression adjusting for age and sex. Hedges’ g reported for continuous variables (T2D − Control).

PS = permeability–surface area product; E = water extraction fraction; CBF = cerebral blood flow

**Table 3:** Associations between BBB permeability, metabolic markers, and cognition.

Predictor	Covariates	β (95% CI)	Partial R^2^	f^2^	P
**PS (mL/100g/min) association with metabolic markers; n = 28**					
HbA1c (%)	Age, Sex, Statin	+ 26.53 (5.30, 47.75)	0.217	0.33	0.011*
Total cholesterol (mg/dL)	Age, Sex, Statin	−0.46 (−0.75, −0.18)	0.331	0.49	0.003*
HDL cholesterol (mg/dL)	Age, Sex, Statin	−0.88 (−1.76, 0.01)	0.154	0.18	0.053*
LDL cholesterol (mg/dL)	Age, Sex, Statin	−0.59 (−0.96, −0.21)	0.313	0.45	0.004*
**PS (mL/100g/min) association with executive function; n = 27**					
D-KEFS Switching	Age	−0.44 (−0.92, 0.04)	0.135	0.16	0.071
D-KEFS Interference	Age	−0.31 (−0.78, 0.17)	0.073	0.08	0.193
TMT-B	Age, Sex, Education	−0.40 (−0.74, −0.05)	0.186	0.23	0.025*
FAS	Age, Sex. Education	−0.39 (−0.74, −0.04)	0.178	0.22	0.029*

* P < 0.05.

The scores are percentile ranks unless otherwise noted.

β, unstandardized coefficient with 95% confidence interval; f^2^, Cohen’s partial effect size (≥ 0.02 small, ≥ 0.15 medium, ≥ 0.35 large).

PS = permeability-surface area product; D-KEFS = Delis-Kaplan Executive Function System; TMT = Trail Making Test; FAS = Controlled Oral Word Association Test.

**Table 4:** Cognitive performance in T2D and control groups.

	Controls (N = 15)^a^	T2D (N = 12)	P	Hedges’ g
**Repeatable Battery for the Assessment of Neuropsychological Status (RBANS)**				
**Individual Tests**				
List Learning	67.0 ± 33.0	29.0 ± 27.4	0.003*	1.20
Story Memory	58.3 ± 27.3	48.0 ± 34.4	0.406	0.33
Semantic Fluency^b^	58.3 ± 34.5	38.3 ± 33.3	0.147	0.57
Digit Span	64.1 ± 25.9	53.6 ± 29.4	0.338	0.37
Coding	71.1 ± 24.0	44.0 ± 30.5	0.020*	0.97
Figure Copy	66.7 ± 23.2	58.0 ± 27.4	0.392	0.33
Picture Naming^b^	60.6 ± 7.1	57.8 ± 20.9	0.655	0.19
Story Recall	69.8 ± 27.5	51.7 ± 30.4	0.122	0.61
Figure Recall	68.5 ± 22.8	58.0 ± 29.4	0.320	0.39
**Domains**				
Immediate Memory	62.7 ± 30.6	35.8 ± 27.8	0.025*	0.89
Delayed Memory	67.5 ± 26.3	52.8 ± 26.5	0.162	0.54
Visuoconstruction/Visuospatial	68.3 ± 19.8	54.0 ± 28.6	0.157	0.58
Language^b^	61.5 ± 27.1	44.3 ± 26.0	0.111	0.63
Attention Index (SS)	111.7 ± 13.6	99.6 ± 15.8	0.048*	0.80
Global Index (SS)	110.7 ± 12.1	96.5 ± 15.7	0.018*	0.99
**Delis-Kaplan Executive Function System (D-KEFS)** ^**c**^				
Color Naming	54.1 ± 25.6	53.8 ± 24.1	0.978	0.01
Word Reading	60.3 ± 18.9	58.5 ± 20.2	0.820	0.09
Switching	82.5 ± 12.5	47.2 ± 33.7	0.006*	1.42
Interference	73.9 ± 15.7	47.1 ± 34.1	0.031*	1.02
**Trail Making Test**				
PartA	61.9 ± 31.8	55.1 ± 31.5	0.582	0.21
Part B	65.9 ± 26.2	44.2 ± 31.0	0.066	0.74
**FAS** ^**b**^	52.9 ± 27.5	33.9 ± 32.4	0.123	0.62
**BNT-30** ^**b**^	44.4 ± 16.0	32.8 ± 24.9	0.176	0.56

* P < 0.05.

Values are mean ± SD. P-values from Welch’s t-test. All scores expressed as normative percentiles adjusted for age (additional adjustments vary by test instrument) unless otherwise noted. SS = scaled score (age-normalized).

Hedges’ g reported as bias-corrected effect size.

One control participant was excluded from all neuropsychological analyses secondary to significant language-based barriers.

An additional control participant, for whom English was a second language, was excluded from language-dependent tests only.

One control participant was unable to complete D-KEFS due to color vision deficiency.

FAS = Controlled Oral Word Association Test; BNT = Boston Naming Test.

## Data Availability

The datasets used in this study are available from the corresponding author upon reasonable request. Please allow reasonable time for response. All the results analyzed supporting the findings of this study are presented within the article.

## Abbreviations

BBB: blood-brain barrier
T2D: type-2 diabetes
ADRD: Alzheimer’s Disease and Related Dementia
PS: permeability-surface area product
HbA1c: Hemoglobin A1c
E: water extraction fraction
CBF: cerebral blood flow
MRI: magnetic resonance imaging
WEPCAST: Water Extraction with Phase-Contrast Arterial Spin Tagging
DCE-MRI: dynamic contrast-enhanced magnetic resonance imaging
PC-MRI: phase-contrast magnetic resonance imaging
SSS: superior sagittal sinus
BMI: body mass index
LDL: low-density lipoprotein
HDL: high-density lipoprotein
HOMA-IR: Homeostatic Model Assessment of Insulin Resistance
MAP: mean arterial pressure
TIV: total intracranial volume
MCI: mild cognitive impairment
AD: Alzheimer’s disease
OLS: ordinary least squares
RBANS: Repeatable Battery for the Assessment of Neuropsychological Status
D-KEFS: Delis-Kaplan Executive Function System
TMT: Trail Making Test
FAS: Controlled Oral Word Association Test
BNT: Boston Naming Test
VTT: venous transit time
GRAPPA: Generalized Autocalibrating Partial Parallel Acquisition
GVS: generalized venous signal
